# Sustained compensatory p38 MAPK signaling following treatment with MAPK inhibitors induces the immunosuppressive protein CD73 in cancer: combined targeting could improve outcomes

**DOI:** 10.1002/1878-0261.13046

**Published:** 2021-07-16

**Authors:** Mikkel G. Terp, Odd L. Gammelgaard, Henriette Vever, Morten F. Gjerstorff, Henrik J. Ditzel

**Affiliations:** ^1^ Department of Cancer and Inflammation Research Institute of Molecular Medicine University of Southern Denmark Odense Denmark; ^2^ Department of Oncology Odense University Hospital Denmark; ^3^ Department of Clinical Research University of Southern Denmark Odense Denmark; ^4^ Academy of Geriatric Cancer Research (AgeCare) Odense University Hospital Denmark

**Keywords:** CD73, MAPKi, p38‐MAPK, RAS‐MAPK, tumor microenvironment

## Abstract

RAS‐MAPK signaling promotes immune evasion and cancer cell survival, and MAPK inhibitors (MAPKis) are frequently used as cancer therapies. Despite progress elucidating the direct effects of MAPKi on immune cells, their indirect effect on the tumor microenvironment (TME) through changes in tumor cells remains incompletely understood. Here, we present evidence of a rapid compensatory response to MAPKi that is driven by sustained p38 MAPK signaling and by which cancer cells can upregulate the immunosuppressive protein CD73 to reduce the antitumor immune response. This compensatory response also results in decreased sensitivity toward MAPKi, and, accordingly, combining anti‐CD73 antibodies and MAPKi significantly enhances the antitumor effect compared to single‐agent treatment *in vivo*. Combining MAPKi and anti‐CD73 was accompanied by significant alterations in intratumor immune cell composition, supporting the effect of MAPKi‐induced CD73 expression on the TME. We show that high CD73 expression significantly correlates with worse outcome in MAPKi‐treated colorectal cancer patients, highlighting the potential clinical importance of increased CD73 expression following MAPKi treatment. Our findings may explain the diminished effect of MAPKi in cancer patients and provides further rationale for combined anti‐CD73 and MAPKi treatment.

## Graphical abstract

RAS‐MAPK signaling promotes immune evasion and cancer cell survival, and MAPK inhibitors (MAPKis) are frequently used in cancer therapy. Here, we present evidence that RAS‐MAPK inhibition increased the expression of the immunosuppressive protein CD73 by a rapid adaptive bypass mechanism involving MAPK‐p38 signaling. Compensatory MAPK‐p38 signaling increased tumor cell growth during MAPKi treatment. Additionally, targeting of CD73 *in vivo* augmented MAPKi. High CD73 expression was associated with a worse outcome in RAS‐MAPKi‐treated patients with colorectal cancer (CRC), indicating that combined anti‐CD73 and MAPKi treatment could be beneficial for patients with CRC resistant to MAPKi therapy.

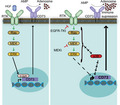

**Abbreviations**
CM: conditioned medium
DUSP: dual‐specificity phosphatase
EGFR: epidermal growth factor receptor
EGFR‐TKI: EGFR tyrosine kinase inhibitor
GR: growth rates
HER2: human epidermal growth factor receptor 2
IFNγ: Interferon γ
MAPKi: MAPK inhibitor
MEKi: MEK inhibitor
MKK: mitogen‐activated protein kinase kinase
PD‐L1: programmed death‐ligand 1
PDX: patient‐derived xenograft
TME: tumor microenvironment

## 1 Introduction

Activating mutations and genomic amplifications of the tyrosine kinase receptors epidermal growth factor receptor (EGFR) and human epidermal growth factor receptor 2 (HER2), and the downstream RAS–mitogen‐activated protein kinase (RAS‐MAPK) pathway, are common in several cancer types, including breast, colorectal, lung cancer, and melanoma, and the clinical benefit of inhibiting this pathway has been thoroughly described [1, 2, 3, 4]. In addition to driving tumor cell proliferation, the RAS‐MAPK pathway plays a central role in the regulation of immune cells within the tumor microenvironment (TME), and dysregulation of this pathway is associated with immune evasion [5, 6]. Acquired resistance to MAPK inhibitor (MAPKi) is a major clinical hurdle caused by mutations affecting inhibitor affinity, nongenomic adaptive bypass mechanisms that transcriptionally upregulate alternative kinase pathways, and activation of kinases downstream of the kinase inhibitors [7, 8, 9]. The activity of kinases within MAPK pathways is tightly regulated, and thus, inhibition of one MAPK pathway might deregulate another. Such adaptive kinome remodeling might also alter the expression of proteins that modulate immune cell functionally within TME. Indeed, preclinical studies in breast cancer and melanoma have shown that while MAPKi primes tumor cells for immune‐mediated killing by upregulating MHC class I expression, the programmed death‐ligand 1 (PD‐L1) immune checkpoint is simultaneously induced and contributes to an immunosuppressive TME [6, 10]. The composition of the immune cells within the TME is of major importance for the response and development of resistance to targeted therapies, such as BRAF and MEK inhibitors (MEKi), as well as blocking antibodies of the EGFR pathway (anti‐EGFR) [6, 10, 11, 12, 13].

CD73 has attracted considerable attention due to its generation of immunosuppressive adenosine [14, 15, 16]. Recently, it was shown that inflammation in melanoma drives CD73 expression through activation of the MAPK (ERK1/2) pathway. CD73 expression was localized and found at sites of ulceration or necrosis, and tissue damage and inflammation following anti‐PD1 treatment also induced CD73 expression [17]. While decreased CD73 expression in MAPKi‐treated patients has been demonstrated, previous reports have only briefly mentioned CD73 upregulation in patients following MAPKi treatment [18]. Furthermore, the cellular factors determining the CD73 expression following MAPKi have not yet been defined nor has the functional consequence of increased CD73 expression on the development of a MAPKi‐resistant TME.

Herein, we propose that a rapid adaptive signaling response following MAPKi administration can direct an increase in expression of the immunosuppressive protein CD73 as well as decrease the sensitivity to the MAPKis in a subset of breast cancer and melanoma patients and in various cell lines from different cancers. In cell lines that upregulate CD73 during MAPKi, we identified compensatory p38 activity as the pivotal driver of CD73 expression and MAPKi resistance. Functionally, the increased CD73 expression following administration of MAPKi was shown to inhibit T‐cell activation, and CD73 blocking antibodies augmented the benefit of MAPKi in *in vivo* mouse models. We also evaluated the clinical significance of CD73 expression in colorectal cancer patients receiving MAPKi (anti‐EGFR, cetuximab) and demonstrated a significantly worse outcome associated with high CD73 expression. Collectively, we show that MAPKi can not only lead to decreased, but also increased, CD73 expression, demonstrating a complex bidirectional regulation of CD73 following MAKPi treatment. Importantly, such CD73 upregulation might be involved in resistance development and diminished response toward RAS‐MAPK pathway inhibition highlighting the potential of anti‐CD73 targeting with current MAPKi, especially in patients with MAPKi‐induced CD73 expression.

## 2 Materials and methods

### 2.1 Cell lines and antitumor agents

The cancer cell lines HCT116, SKBr3, CT26.CL25 (CT26), and A549 were purchased from ATCC. PC9 cells were kindly provided by Roche with the authorization of Dr. Mayumi Ono (Kyushu University, Fukuoka, Japan). MC38 cells were purchased from Kerafast. 4T1.2 (4T1) cells were a kind gift from Dr. Laurence Zitvogel. HCT116 and SKBr3 cells were cultured in McCoy’s 5A medium supplemented with fetal bovine serum (FBS) and penicillin/streptomycin (P/S). PC9, 4T1, and A549 were grown in RPMI supplemented with FBS and P/S. CT26 were cultured in RPMI‐1640 supplemented with 10% FBS, 2 mm Glutamine, 1,5 g·mL^−1^ sodium bicarbonate, 4,5 g·L^−1^ glucose, 1 mm sodium pyruvate, 10 mm HEPES, 0,1 mm nonessential amino acids, and 0,4 mg·mL^−1^ G418 geneticin, and MC38 were cultured in Dulbecco’s modified Eagle’s medium—high glucose supplemented with 10% FBS, 0,1 mm nonessential amino acids, 2 mm Glutamax, 1 mm sodium pyruvate, 10 mm HEPES, 50 μg·mL^−1^ gentamycin, and 1% P/S. To reduce variability between experiments, cells were maintained at low passages numbers (< 10 passages) throughout the experiments. Cells passaged for more than 6 months after receipt or resuscitation from cell bank were authenticated using Cell ID System (Promega, Nacka, Sweden) or IDEXX BioAnalysis before the described experiments to ensure consistent cell identity. All cell lines were frequently tested for mycoplasma (MycoAlert®, Mycoplasma detection kit, Lonza, Copenhagen, Denmark). If not otherwise stated, the concentrations of the following inhibitors were used: EGFR tyrosine kinase inhibitor (EGFR‐TKI, gefitinib, ZD1839, 100 nm), (MEKi, trametinib, GSK1120212, 100 nm), Akt inhibitor (Akti, capivasertib, AZD5363, 2.5 μm), p38 inhibitor (p38i, SB203580, 10 μm and SB202190, 10 μm), and PI3K inhibitor (PI3Ki, alpelisib, BYL‐719, 1 nm) were purchased from Selleck Chemicals (Berlin, Germany). Naphthol‐AS‐E (100 nm) was purchased from Merck Life Science (Soeborg, Denmark).

### 2.2 Western blot

Cells were washed in ice‐cold Tris‐buffered saline (TBS) and lysed in RIPA (10 nm Tris/HCL, pH 8, 5 mm Na_2_EDTA, pH 8, 1% NP‐40, 0.5% sodium deoxycholate, 0.1% SDS), supplemented with phosphatase and protease inhibitors (Roche, Hvidore, Denmark, #116995001, #1183670001). Protein (5–40 μg) was loaded on 4%–12% Mini Proteam TGX gels (Bio‐Rad, Copenhagen, Denmark, # 456‐1096) transferred onto PVDF membrane using Trans‐Blot Turbo RTA Transfer Kit, PVDF [(Bio‐Rad, #1704272), blocked, and incubated with primary antibodies against phospho‐p38 (Thr180/Tyr182) #9211], p38 (#9212), phospho‐p44/42 MAPK (Erk1/2) (#4370), p44/42 MAPK (Erk1/2) (#9102), cyclin D1 (#2978), c‐JUN (#9165), and alpha‐tubulin (#2148), all from Cell Signaling Technology (Leiden, the Netherlands) and β‐actin (ab6276, Abcam, Cambridge, UK) at 4 °C. Thereafter, the membranes were incubated with goat anti‐rabbit (Dako, Glostrup, Denmark, #P0448) or goat anti‐mouse HRP‐conjugated secondary antibodies (Dako #P0447) at room temperature (RT). Western blots were developed using Clarity^TM^ western ECL Substrate (Bio‐Rad, #170‐5060) and CL‐Xposure film (Thermo Fisher Scientific, Roskilde, Denmark, #34089).

### 2.3 RT‐PCR

RT‐PCR was performed as previously described [19]. Briefly, total RNA was purified using Isol‐Lysis Reagent, TRIzol (Thermo Fisher Scientific). cDNA synthesis was performed using RevertAid Premium Reverse Transcriptase Kit (Thermo Fisher Scientific). The relative quantification of gene expression was performed using SYBR Green PCR Master Mix (Thermo Fisher Scientific) according to the manufacturer´s instructions. All primers were purchased from Qiagen (Copenhagen, Denmark). PUM1 and HPRT was used as reference gene for normalization for the human and mouse samples, respectively. The relative expression levels were calculated using the comparative threshold method [20].

### 2.4 Cell viability assays

Cells (1–2 x 10^4^) were seeded in 24‐well plates and treated with MAPKi [EGFR‐TKI (100 nm) or MEKi (100 nm)] or p38i (10 μm) alone or in combination. At indicated times, cell growth was evaluated using crystal violet staining. Cells were washed twice in PBS and stained by incubation with 0.5% crystal violet in 25% V/V Methanol (Merck Life Science, # V5265) 25% V/V Methanol for 10 min. Following a thorough wash in H_2_O to remove the Crystal violet solution, the stained cells were dissolved in citrate buffer (0.1 mm sodium citrate in 50% EtOH) while shaking for 30 min at RT. Absorbance at 570 nm were measured using a PARADIGM (Beckman Coulter, Copenhagen, Denmark).

### 2.5 siRNA knockdown

Cells were seeded in 6‐well plates and MAPK14 siRNA (Qiagen) was transfected using Lipofectamine 3000 reagent according to the manufacturer’s guidelines (Thermo Fisher Scientific). Knockdown was evaluated after 48 and 72 h by RT‐PCT. Twenty‐four hours after siRNA transduction, cells were treated with MEKi and CD73 expression was analyzed after 72 h of treatment.

### 2.6 CD73 enzymatic activity

Enzymatic activity was measured as previously described [21] by measuring produced inorganic phosphate, equivalent to the conversion of AMP to adenosine. In brief, cells exposed to drugs for the indicated times (5 × 10^4^) were incubated with and without 0.2 mm AMP for 30 min at 37 °C. The generated inorganic phosphate was detected using the phosphate reaction solution (0.4% NH_4_‐molybdate, 10% ascorbic acid) in the presence of H_2_SO_4_. The color‐reaction product (molybdenum blue) was colorimetrically measured at 560 nm using a Victor3 Multilabel Plate Reader (PerkinElmer, Skovlunde, Denmark). Catalytic activity was determined by subtracting the values in the absence of from the values in the presence of AMP.

### 2.7 Flow cytometry

Cells were seeded and immediately treated with drugs for the indicated times. Following treatment, cells were harvested with EDTA (5 mm). HCT116, A549, SK‐Br‐3, and PC9 cells were incubated with anti‐human CD73 antibody (mouse anti‐human AD2, a gift from Prof. L. Thompson, Oklahoma Medical Research Foundation, Oklahoma City, OK, USA), while 4T1, CT26, and MC38 were incubated with anti‐murine CD73 antibody (rat anti‐mouse TY/23, a kind gift from Dr. Linda Thompson) on ice. Following 3x wash in cold FACS buffer (PBS, 1% BSA), the cells were incubated in either Alexa Fluor 488 anti‐mouse (Thermo Fisher Scientific, #A11029) or Alexa Fluor 647 anti‐rat secondary antibody (Thermo Fisher Scientific, #A21247). The cells were washed 3x and resuspended in FACS buffer containing either TO‐PRO‐3 (1 : 2000, Thermo Fisher Scientific, #T3605; AD2 stained cells) or SytoxBlue (1 : 1000, Thermo Fisher Scientific, #S34857; TY/23‐stained cells) to visualize dead cells. The cells were analyzed on a LSRII (BD Biosciences, Albertslund, Denmark).

Single‐cell suspensions of mouse tumor cells were stained with Brilliant Violet 605 anti‐mouse CD45 (BioLegend, Nordic Biosite, Copenhagen, Denmark, #103139) and PerCP anti‐mouse CD3ɛ (BioLegend, Nordic Biosite, #100325) and counterstained with the LIVE/DEAD fixable Near‐IR cell death marker (Thermo Fisher Scientific). The cells were analyzed on The BD *FACSAria*™ III (BD Biosciences). Data were analyzed using flowjo software (V.10, FlowJo, Ashland, OR, USA).

### 2.8 T‐cell activation assay

4T1 cells were seeded in 6‐well plates and exposed to MEKi or control medium for 72 h. Following drug exposure, cells were harvested and 1 x 10^5^ cells were incubated in medium containing 0.2 mm AMP (Merck Life Science, #A1752) with or without anti‐CD73 antibody TY/23. Following 10 min incubation, conditioned medium (CM) was harvested and applied to T cells purified from PBMCs using a T cell purification kit according to the manufacturer’s guidelines (Thermo Fisher Scientific, #88‐7316). After 30 min, the T cells were activated using anti‐CD3/CD28 beads (Thermo Fisher Scientific, #11161D). Following 96 h incubation, supernatants were harvested and IFNg secretion was measured using an ELISA kit (Thermo Fisher Scientific, #11344D) according to the manufacturer’s guidelines.

### 2.9 *In vivo* studies

Murine 4T1 cells (1 x 10^5^) were injected into the 4th right mammary fat pad of female Balb/cJBomTac mice (Taconic Biosciences GmbH). Mice (*n* = 8 per group) were treated with either (a) anti‐CD73 antibody alone (100 mg, clone TY/23, at days 5, 9, and 14), (b) trametinib alone (1 mg·kg^−1^, daily), (c) anti‐CD73 antibody and trametinib combined, or (d) vehicle control. Tumor volume was calculated as follows: tumor volume = 0.5 x (length) x (width)^2^. All animal experiments were approved by the Experimental Animal Committee of The Danish Ministry of Justice and were performed at the animal core facility at the University of Southern Denmark. Animals were euthanized if they showed any adverse signs of disease, including weight loss, paralysis, thymus dysfunction, or general discomfort. Mice were housed under pathogen‐free conditions with *ad libitum* food and water.

### 2.10 Immunofluorescence

*Cells were seeded at low densities* and subsequently treated with drugs for 72 h. Cells were fixed in 4% formaldehyde, permeabilized in 0.05% Triton X‐100, and stained with an anti‐Ki67 antibody (Thermo Fisher Scientific, #RM9106). The cells were mounted in ProLong^TM^ Gold Antifade Reagent with DAPI (Thermo Fisher Scientific).

### 2.11 Dissociation of tumors

Tumors were harvested and minced completely into small pieces using scalpels followed by incubation for 45 min in 10ml of Dissociation buffer (2 mg·mL^−1^ collagenase IV (Merck Life Science, #C5138)) supplemented with 4 U·mL^−1^ DNAse I (Thermo Scientific, #EN0521), with constant low agitation at 37 °C. The cell suspension was pipetted up and down for 2 min using a 5 mL pipette and washed in DPBS supplemented with 5% FBS (complete DPBS, 10 min at 300 x g). The cell suspension was filtered using a 70 μm Cell Strainer (BD, # 352350), and, following centrifugation at 300 g for 10 min, the cells were resuspended in 2 mL of Red Blood Cell Lysing buffer (155 mm NH_4_Cl, 12 mm NaCO_3_, 0.1 mm EDTA) and gently mixed for 1 min at RT. The cell suspension was washed twice in 20 mL of complete DPBS, resuspended in 5 mL of complete DPBS, and filtered using a 70 μm Cell Strainer. The single cells were counted and stained for flow cytometry.

### 2.12 Statistical analysis

Statistical comparison of expression levels in flow cytometry analysis, enzymatic activity, and gene expression between different drug exposures was performed using either a two‐tailed Student’s *t*‐test with Bonferroni post hoc test for adjustment of multiple comparison or one‐way ANOVA with Bonferroni multiple comparison test, as indicated. Tumor size was compared using the linear mixed effects model with categorical treatments groups and continuous time as fixed effects including the interaction. The model contains a random effect for the individual tumors to take repeated measurements within each mouse into account. The group specific time slope is referred to as growth rates (GR), and the reported p‐values represent the difference in GR of the combined anti‐CD73 antibody and MEKi compared to control or either treatment alone. *P*‐values < 0.05 were considered statistically significant (*P < 0.05, **P < 0.01, ***P < 0.001, and ****P < 0.0001). Statistical analyses were performed using graphpad prism version 8 (GraphPad Software, San Diego, CA, USA).

### 2.13 Patients and survival analysis

A study including patients with metastatic colorectal cancer receiving cetuximab (anti‐EGFR) monotherapy was available from the GEO public database with the accession number GSE5851. Patients with metastatic colorectal cancer with at least one prior chemotherapeutic regimen for advanced disease completed at least 4 weeks before enrollment or who had refused prior treatment were eligible. A pretreatment tumor biopsy was taken from the metastatic site before receiving standard cetuximab treatment. Seventy patients with known KRAS mutation status were included in the survival analysis [22]. As described previously [23], progression‐free survival (PFS) was estimated by the Kaplan–Meier method and the nonparametric log‐rank test was applied to compare the different groups [24]. To define the optimal cutoff for CD73 expression, we applied a ‘cutoff finder’ as reported by Budczies *et al*. [25], which uses a log‐rank statistical test to determine the cut point, and a cutoff value of 8.625 was identified to divide patients into two groups. The Cox multivariate regression model was applied with CD73 expression as covariate, obtaining hazard ratio and 95% confidence interval (CI). Significance levels of < 0.05 in the univariate model were used to select variables for the Cox multivariate regression model.

Each analysis was performed using a two‐tailed 5% significance level and a 95% CI. A two‐tailed Student’s t‐test was used to compare CD73 mRNA expression between responders (partial response and stable disease) and nonresponders (stable disease and undetermined). Statistical analyses were performed using stata version 15 (Statacorp, College Station, TX, USA) and graphpad prism version 8 (GraphPad Software).

### 2.14 CD73 expression analysis using publicly available datasets

Expression data from before and during BRAF‐targeted treatment (dabrafenib or vemurafenib) in melanoma patients (*n* = 8) was available from the GEO public database, accession number GSE99898. Pre‐ and post‐trastuzumab (anti‐HER2)‐treated breast cancer patients (*n* = 17) were available from the GEO public database, accession number GSE114082. The data from both patient cohorts were analyzed using the interactive GEO2R web tool to determine gene expression across experimental conditions. GEO2R analyzes original submitter‐supplied processed data tables using the GEOquery and limma R packages from the Bioconductor project. The distribution of values for the selected samples is presented in Fig. S1A‐B, and median‐centered values indicate the data are normalized and cross‐comparable. Fold‐change values were calculated as the ratio between before and during sample signal values. Expression data from vehicle and acute and sustained trametinib‐treated pancreatic adenocarcinoma PDX models (*n* = 2) were available from the GEO public database, accession number GSE98399. Data were analyzed using the Transcriptome analysis console program (Affymetrix).

## 3 Results

### 3.1 Upregulation of CD73 expression and enzymatic activity in cancer cells upon inhibition of the RAS‐MAPK pathway

**Fig. 1:**
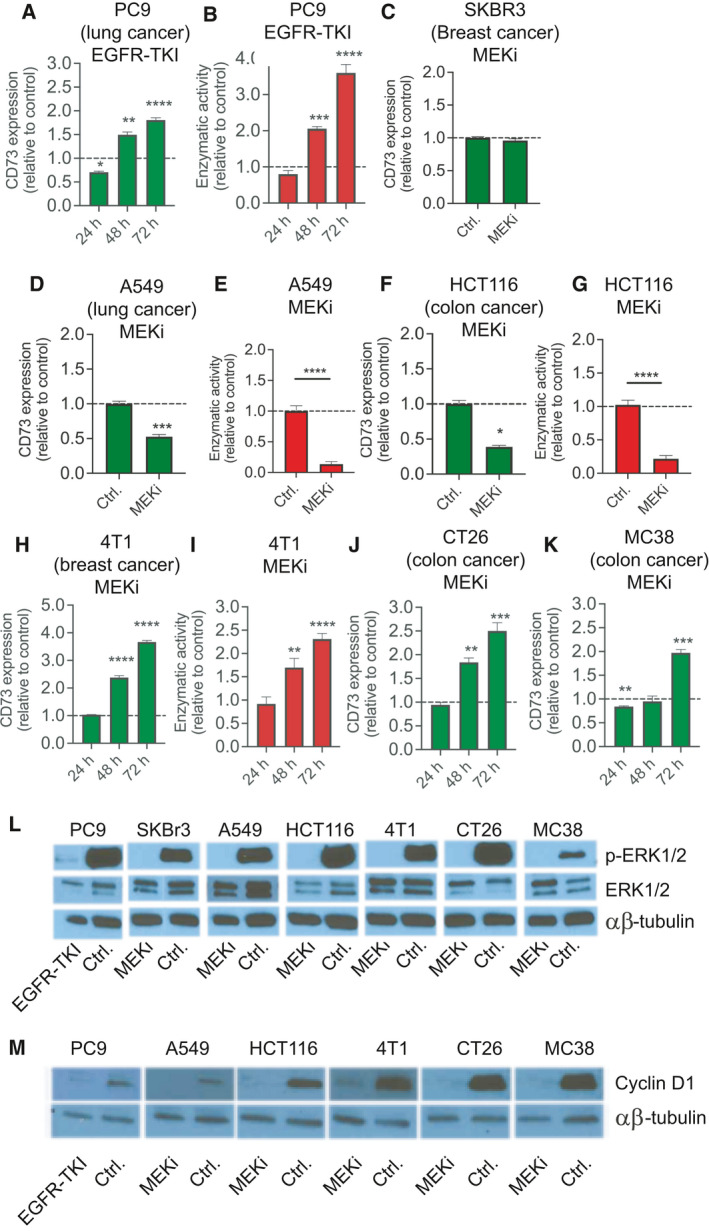
CD73 expression is increased in a subset of tumor cell lines following RAS‐MAPK pathway inhibition. (A–B) PC9 cells were exposed to EGFR‐TKI for the indicated time periods, and (A) CD73 protein expression (green columns) and (B) enzymatic activity (red columns) were subsequently measured. (C) SKBr3, (D–E) A549, and (F–G) HCT116 cells were exposed to MEKi for 72 h, and CD73 protein expression (green columns) and enzymatic activity (red columns) were subsequently measured. (H–I) Murine 4T1, (J) CT26, and (K) MC38 cells were exposed to MEKi for the indicated times, and CD73 protein expression (green columns) and enzymatic activity (red columns) were subsequently measured. (L) Western blot analysis of ERK1/2 and pERK1/2 proteins following gefitinib or MEKi exposure for 72 h. (M) Western blot analysis of cyclin D1 protein following gefitinib or MEKi exposure for 72 h. In all experiments, EGFR‐TKI and MEKi were used at 100 nm each. Results shown are mean ± SD of experiments performed in triplicates and representative of three independent experiments. *Asterisks* indicate significant differences in two‐tailed *t*‐test test for the drug‐exposed vs. untreated control cells at the same time point (*P < 0.05, **P < 0.01, ***P < 0.001, and ****P < 0.0001).

To investigate the molecular mechanisms leading to CD73 up‐ or downregulation following MAPKi treatment, we initially investigated alterations in CD73 expression in a panel of human and mouse cancer cell lines following relevant RAS‐MAPK‐targeted treatment, including MEK inhibitor (MEKi, trametinib) and EGFR‐TKI (gefitinib). In the human EGFR‐mutant NSCLC cell line PC9, CD73 expression and its enzymatic activity was initially decreased following exposure to EGFR‐TKI but reverted after 48 h to a significant increase in CD73 expression up to 72 h (Fig. 1A,B, *P* < 0.05). When PC9 were treated with MEKi, we also observed an increase of CD73, albeit slightly lower (Fig. S2A). In contrast, following MEKi exposure for 72 h in the human HER2‐amplified breast cancer cell line SKBr3, CD73 expression remained unchanged (Fig. 1C), while we consistently observed significant downregulation of CD73 expression and decreased enzymatic activity upon MEKi exposure in the human NSCLC cell line A549, and the human colon cancer cell line HCT116 (Fig. 1D–G). To address how increased CD73 expression following MEKi treatment affected the interplay with the immune system *in vivo*, we also tested mouse mammary tumor‐derived cell lines that could readily be transplanted into immunocompetent syngeneic mice. In the breast cancer cell line 4T1, a time‐dependent increase in CD73 expression was observed, similar to the PC9 cells, and after 72 h exposure, the expression and enzymatic activity of CD73 increased by 3.6‐ and 2.3‐fold, respectively (*P* < 0.05, Fig. 1H,I). A comparable MEKi‐induced CD73 upregulation was also demonstrated in the two murine colon cancer cell lines, CT26 and MC38 (Fig. 1J,K). Interestingly, the regulation of CD73 following RAS‐MAPKi did not seem to correlate with the baseline expression of CD73 in the cell lines (Fig. S2B). To assure that the altered CD73 expression was not due to bypassing and re‐activation of the RAS‐MAPK pathway, levels of the downstream target ERK1/2 were evaluated in all cell lines after 72 h exposure to MAPKi. Indeed, MEKi and gefitinib treatment showed complete inactivation of ERK1/2 (Fig. 1L). As an additional control for effective pathway inhibition, we analyzed the expression of cyclin D1, a transcriptional target of the RAS‐MAPK pathway known to be negatively regulated by MAPKi exposure [26, 27]. As expected, cyclin D1 expression decreased following RAS‐MAPK pathway inhibition compared to controls for PC9, 4T1, CT26, MC38, A549, and HCT116 (Fig. 1M). Taken together, these data show that in the majority of cancer cell lines, upregulated or unchanged CD73 expression occurs after effective inhibition of the MAPK‐RAS pathway, while in the remaining cancer cell lines, the CD73 levels decreased. Inhibition of other major signaling pathways, including PI3K‐AKT and JNK pathways, did not consistently lead to increased CD73 expression in the same models (Fig. S2C).

### 3.2 Increased CD73 expression in tumor samples of patients treated with MAPKi

**Fig. 2:**
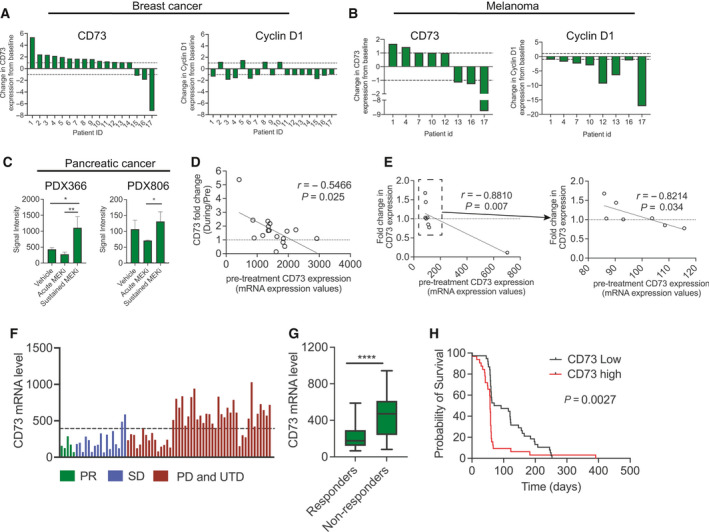
CD73 is upregulated in a subset of breast cancer, melanoma patients, and pancreatic PDX models during RAS‐RAF‐MEK inhibitory treatment and is associated with a worse outcome following cetuximab in colorectal cancer patients. (A) CD73 and cyclin D1 gene expression was analyzed in publicly available datasets (GSE114082) of 17 matched before and during treatment samples from breast cancer patients treated with trastuzumab. (B) CD73 and cyclin D1 gene expression was analyzed in publicly available datasets (GSE99898) of 8 matched before and during treatment samples from melanoma patients treated with BRAF inhibitors alone or in combination with MEKis. (C) Analysis of publicly available gene expression data from two pancreatic cancer PDX models (PDX366 and PDX806—GSE98399). This dataset consisted of samples taken from acute MEKi (24 h trametinib treatment), sustained MEKi (3 months of trametinib treatment) and control treatment. *Asterisks* indicate significant differences in unpaired *t*‐test for the acute MEKi vs. sustained MEKi treatments (*P < 0.05 and **P < 0.01). The expression of CD73 is presented as the signal intensity output from the gene array. Results shown are mean ± SD of the analysis of triplicates from a single tumor. (D–E) Correlation between CD73 expression in the pre‐treatment samples and its alteration by RAS‐MAPK inhibition (fold change) in the (D) breast cancer patients (GSE114082) and in the (E) melanoma patients (GSE99898). For the melanoma patients (E), the right graph represents the data with the outlier point excluded (dotted box). Spearman coefficients and relative *P*‐values are shown. (F) CD73 gene expression was analyzed in a cohort of 70 metastatic colorectal cancer patients from a publicly available dataset (GSE5851). Patients are ordered based on best clinical responses [partial response (PR, *n* = 5), stable disease (SD, *n* = 17), progressive disease (PD, *n* = 37), and patients with undetermined responses (UDT, *n* = 11)]. Dotted line defines cutoff between CD73^high^ and CD73^Low^ as defined by a ‘cutoff finder’ as reported by Budczies *et al*. [25] (G) A significant difference in CD73 gene expression (****P < 0.0001, two‐tailed Student’s *t*‐test) was observed between responders (PR and SD) and nonresponders (PD, *n* = 37). (H) Kaplan–Meier plots showing the correlation between CD73 expression and progression‐free survival of the same 70 cetuximab‐treated metastatic colorectal cancer patients.

We then evaluated whether CD73 upregulation was also observed in clinical tumor samples from patients treated with MAPKi. We analyzed paired primary HER2‐positive breast cancer samples from a cohort of 17 patients obtained before and during treatment with single‐agent neoadjuvant trastuzumab (GSE114082). HER2 in breast cancer is a relevant RAS‐MAPK pathway target. Furthermore, we observed steady CD73 expression in SKBr3 following inhibition of MEKi as opposed to the previously reported decrease following inhibition of the RAS‐MAPK pathway. We found that 53% (9/17) exhibited increased CD73 expression (1.6‐ to 5.3‐fold), while CD73 was decreased in 12% (2/17) of tumors (0.5‐ to 0.14‐fold) and unchanged in 35% (6/17) (0.82‐ to 1.23‐fold) (Fig. 2A). The expression patterns of cyclin D1 differed substantially from CD73, and among the 9 patients with increased expression of CD73, cyclin D1 was downregulated or unchanged in 8 patients, confirming that the MAPK‐RAS pathway was effectively inhibited (Fig. 2A).

In addition, we also analyzed the gene expression data from a second cohort of matched tumor samples of 8 melanoma patients obtained prior to and during treatment with the BRAF inhibitors dabrafenib or vemurafenib alone or in combination with the MEKi trametinib (GSE99898) (Fig. 2B). In this dataset, 25% of tumors exhibited CD73 upregulation (≥1.5‐fold), while only one tumor (12.5%) exhibited a substantial decrease in CD73 expression. The remaining 5 tumors (62.5%) showed unchanged CD73 expression (Fig. 2B). Moreover, for the majority of these patients, cyclin D1 expression was downregulated (Fig. 2B). We also evaluated publicly available gene expression data from two pancreatic cancer patient‐derived xenograft (PDX) models (PDX366 and PDX806, GSE98399). CD73 expression decreased acutely (24 h) after MEKi treatment but was upregulated following sustained treatment for 3 months and significantly increased compared to acute MEKi treatment in PDX366 and PDX806 (Fig. 2C). Taken together, these data suggest that the adaptive signaling response that drives CD73 expression in tumors following efficient MAPKi treatment is also present in cancer patients.

In analyzing the change in CD73 expression following RAS‐MAPK inhibition, an inverse correlation between the level of CD73 in the pretreatment biopsies and the induction following treatment was observed (breast cancer samples: *r* = −0.5466, *P* = 0.025; melanoma samples: *r* = −0.8810, *P* = 0.007) (Fig. 2D,E). In the melanoma samples, the correlation is also significant when the outlier point is excluded (*r* = −0.8214, *P* = 0.034, Fig. 2E). These data indicate that CD73 is mostly induced in tumors with a lower basal level of CD73 expression before treatment. Nevertheless, the cell line studies of 4T1 and PC9 demonstrate that CD73 can also be induced in cells which already have high CD73 expression (Fig. S2B).

### 3.3 CD73 expression correlates with clinical outcome in anti‐EGFR‐treated colorectal cancer patients

Next, we examined whether the response to MAPKi treatment in patients correlated with the CD73 expression level. To this end, analyzing the effect of CD73 expression on patient outcome following MAPKi could provide indications of a potential scenario where the effect of MAPKi‐targeted treatment could be hampered by a potential induction of CD73 by the treatment itself. To address this clinical question, we evaluated the correlation between clinical outcome on anti‐EGFR treatment and tumor CD73 expression using a publicly available dataset of 70 cetuximab‐treated colorectal cancer patients (GSE5851). Initially, we divided the tumors into CD73^high^ and CD73^low^ groups based on their expression levels using a ‘cutoff finder’ that uses log‐rank statistical test to determine the cut point [25]. Subsequently, patients were stratified as either responders [total *n* = 22, 4 partial responders (PR) and 17 with stable disease (SD)] or nonresponders [total *n* = 47, 37 patients with progressive disease (PD) and 11 patients with undetermined responses (UTD) due to death before their first radiographic assessment]. Importantly, all tumors of patients with PR and 15 of 17 patients with SD exhibited low CD73 levels (Fig. 2F) and CD73 expression was significantly lower in tumors of responders vs. nonresponders (Fig. 2G, *P* < 0.0001). Further, the median PFS was significantly lower in CD73^high^ patients [57 days (95% CI, 49–59)] compared to CD73^low^ patients [70 days (95% CI, 60–122)] (*P* = 0.0027, nonparametric log‐rank test, Fig. 2H). Univariate Cox proportional hazards regression analysis showed that the predictive value of CD73 expression was independent of KRAS mutational status (Table S1). This analysis indicates that high CD73 expression is associated with a poor response to anti‐EGFR treatment and suggests that high CD73 expression, which potentially could be further induced by the cetuximab treatment, may be involved in the development of tumor resistance during treatment.

### 3.4 Sustained p38 activation induces CD73 expression following RAS‐MAPK pathway inhibition

**Fig. 3:**
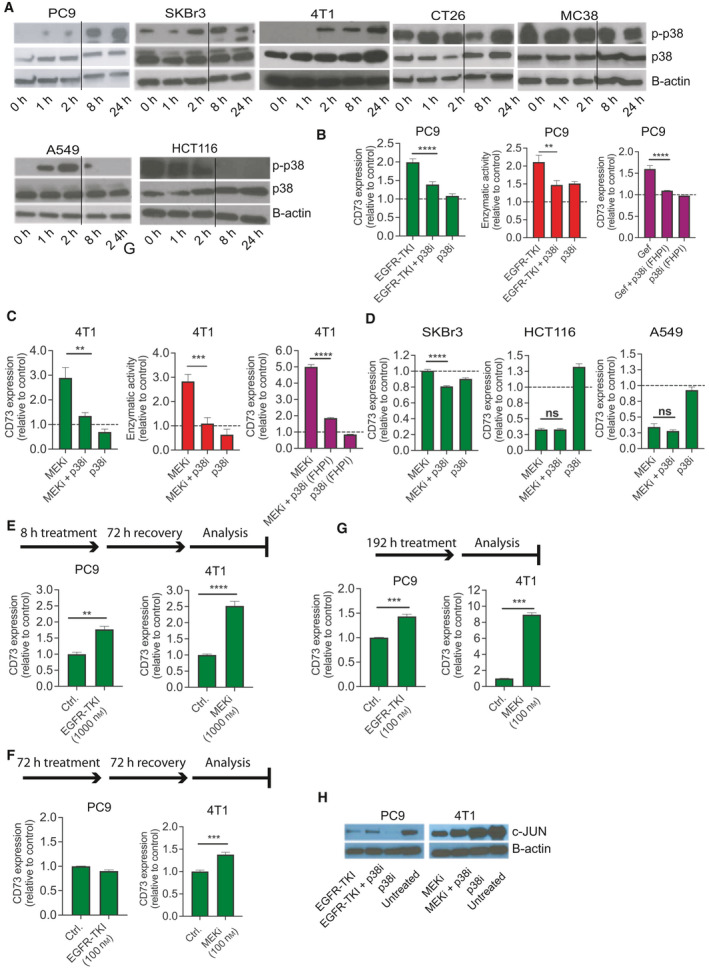
Upregulation of CD73 expression is driven by sustained p38 activation (A) PC9 were exposed to EGFR‐TKI (100 nm), while SKBr3, 4T1, CT26, MC38, A549, and HCT116 were exposed to MEKi (100 nm). Western blot analysis of phosphorylated p38 (p‐p38) and p38 protein following drug exposure for the indicated time points. The Western blots was repeated with similar results. The quantification of the presented blot is shown in Suppl. Fig. 3. (B–C) PC9 and 4T1 cells were exposed to EGFR‐TKI or MEKi and the p38‐MAPK inhibitor (SB203580) (p38i; green columns) or the p38‐MAPK inhibitor (SB202190; p38i (FHPI; purple columns) alone or in combination for 72 h, at which time point CD73 expression was analyzed by flow cytometry, or the enzymatic activity was determined by measuring the generated inorganic phosphate from the enzymatic cleavage of AMP (red bars). (D) SKBr3, HCT116, and A549 cells were exposed to MEKi and p38i, alone or in combination for 72 h, at which time point CD73 expression was analyzed by flow cytometry. (E) PC9 and 4T1 were exposed to EGFR‐TKI (1000 nm) or MEKi (1000 nm), respectively, for 8 h following subsequent incubation without drugs for 72 h. CD73 expression was analyzed by flow cytometry. (F) PC9 and 4T1 were exposed to EGFR‐TKI or MEKi, respectively, for 72 h following subsequent incubation without drugs for 72 h. CD73 expression was analyzed by flow cytometry. (G) PC9 and 4T1 were exposed to EGFR‐TKI or MEKi, respectively, for 192 h. CD73 expression was analyzed by flow cytometry. (H) Western blot analysis of c‐JUN expression in PC9 and 4T1 cells exposed to EGFR‐TKI or MEKi in combination with p38 inhibitor for 72 h. Results are shown as mean ± SD and represent three replicates. *Asterisks* indicate significant differences in one‐way ANOVA test with Bonferroni multiple comparison test for the drug combination‐treated cells compared to cells treated with either drug alone (**P < 0.01, ***P < 0.001, and ****P < 0.0001).

To evaluate the underlying mechanism causing CD73 upregulation in cancer cells in response to effective RAS‐MAPK inhibition, we examined p38 activation, as shown by rapid phosphorylation [28] (Fig. 3A,B). Sustained p38 activation for more than 24 h was observed in the cell lines 4T1, PC9, CT26, MC38, and SKBr3, all of which exhibited upregulation or steady expression of CD73 in response to RAS‐MAPK inhibition, while the initial p38 activation in HCT116 and A549 cells was abolished after 2 h and 4 h, respectively (Figs 3A and S3). The latter two cell lines exhibited downregulation of CD73 in response to RAS‐MAPK inhibition. These data suggest that sustained activation of p38 may be a driver of CD73 upregulation.

Next, we examined whether p38 activation was responsible for CD73 upregulation in response to inhibition by MAPKi using the p38 inhibitor SB203580 (p38i). We found that inhibition of p38 significantly reduced MAPKi‐induced CD73 upregulation in PC9, 4T1, CT26, and MC38 (Figs 3B,C and S4A‐B). The MAPKi‐induced increase in enzymatic activity in 4T1 and PC9 was also significantly reduced in response to p38 inhibition (Fig. 3B,C). We also tested a second p38i [SB202190 (p38i‐FHPI)]. Similar to p38i (SB203580), we observed that p38i‐FHPI (SB202190) significantly reduced MAPKi‐induced CD73 upregulation in 4T1 and PC9 cells (Fig. 3B,C). In SKBr3, steady CD73 expression in response to MEKi alone was actually significantly decreased when MEKi and p38i were combined compared to MEKi alone at both protein and mRNA levels (Figs 3D and S4C). This suggests that p38 activation prevents MEKi‐induced decrease in CD73 expression although p38 activation cannot drive CD73 upregulation. No effect of p38 inhibition was observed in A549 and HCT116 in which p38 was not sustainably activated (Figs 3D and S4C). Both p38i inhibitors specifically block both p38α and p38β, but not p38δ and p38γ. Interestingly, when using ralimetinib, an inhibitor that only blocks p38α, we observed only a slight, but significant, reduction in MAPKi‐induced CD73 upregulation in PC9 and no effect in 4T1 (Fig. S4D). Additionally, effective knockdown of p38α (MAPK14) in 4T1 (Fig. S4E) showed no effect on MAPKi‐induced CD73 upregulation (Fig. S4F). Efficient knockdown of p38α in PC9 was not observed (Fig. S4E). Collectively, this supports a role for both p38α and p38β in the upregulation of CD73, and when only p38α is inhibited, p38β continues to drive the signaling pathway.

**Fig. 4:**
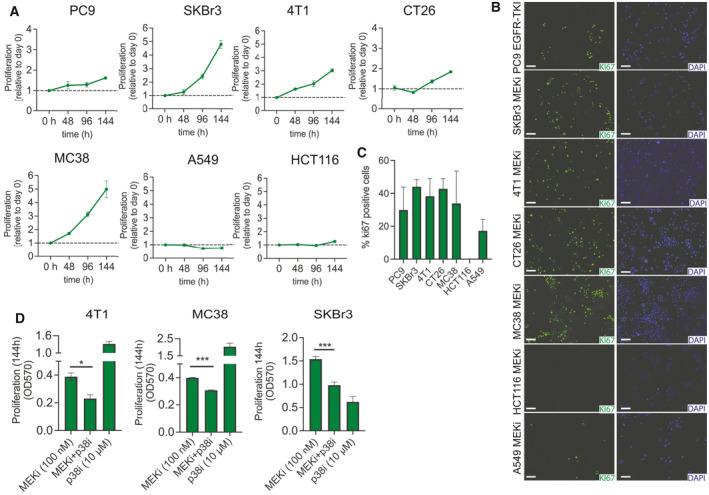
Activation of p38 decrease sensitivity toward MAPKi. (A) Cells were seeded at low density. PC9 was exposed to EGFR‐TKI (100 nm), and SKBr3, 4T1, MC38, CT26, A549, and HCT116 were exposed to MEKi (100 nm). Tumor cell growth was determined at the indicated times using crystal violet staining and normalized to that of day 0. (B) PC9 was exposed to EGFR‐TKI (100 nm), and SKBr3, 4T1, MC38, CT26, A549, and HCT116 were exposed to MEKi (100 nm). Following 72 h of treatment, cells were stained for Ki67 expression. (C) The percentage of Ki67‐positive cells was quantified using ImageJ. Results shown are mean ± SD of experiments performed in triplicates (D) 4T1, MC38, and SKBr3 were seeded at low density and exposed to MEKi or p38i alone or in combination. Tumor cell growth was determined after 144 h for 4T1 and MC38 and after 72 h for SKBr3. Results shown are mean ± SD of experiments performed in triplicates. *Asterisks* indicate significant differences in one‐way ANOVA test with Bonferroni multiple comparison test for the drug combination‐treated cells compared to cells treated with either drug alone (*P < 0.05, **P < 0.01, and ***P < 0.001).

We next investigated the rate and durability of MAPKi‐induced CD73 expression. When 4T1 and PC9 cells were incubated with a high dosage of MEKi or EGFR‐TKI (1000 nm) for only 8 h following 72 h of incubation without MAPKi, we also observed a significant increase in CD73 expression (Fig. 3E). However, if cells were incubated with a lower dose of MAPKi (100 nm) for 72 h, CD73 expression was almost normalized following a subsequent incubation without MAPKi for 72 h (Fig. 4F). If the selective pressure of MAPKi was maintained for 192 h, CD73 expression in PC9 was stabilized to the level observed following 72 h of incubation, while CD73 was even further increased in 4T1 compared to the level after 72 h exposure (Fig. 4G). This suggests that MAPKis induce a rapid, but reversible activation of a signaling cascade that can drive CD73 expression.

Previous studies have shown that p38 can activate the AP1 transcription complex member c‐JUN [29, 30], and indeed, c‐JUN has also been shown to coordinate MAPK‐driven CD73 transcription [17]. However, we found that the expression of c‐JUN was either decreased or unchanged in PC9, 4T1, CT26, and MC38 following MAPKi exposure (Figs 3H and S4G), suggesting that CD73 upregulation is not due to p38‐mediated activation of c‐JUN, but rather a transcriptional regulator other than the one coordinating MAPK‐driven CD73 expression. Since Loi *et al*. [31] previously showed that CD73 is upregulated following chemotherapy in a CREB‐dependent manner, we investigated whether inhibition of CREB by Naphthol‐AS‐E could reverse the effect of MEKi exposure on CD73 upregulation, but no such effect was observed (Fig. S4H).

### 3.5 Sustained activation of p38 that upregulates CD73 expression might also decrease the sensitivity toward RAS‐MAPK pathway inhibition

Next, we evaluated whether induction of MAPK‐p38 signaling affected the sensitivity toward the MAPKi treatment. Upon exposure of the RAS‐MAPK inhibitor at the same concentration used in the CD73 expression analysis, proliferation was decreased in all the cell lines at varying degrees (Fig. S5A). Strikingly however, the cell lines 4T1, MC38, CT26, PC9, and SKBr3, where CD73 was upregulated or steady, continued to grow in the presence of the inhibitors, while HCT116 and A549 cell growth was completely inhibited (Fig. 4A). Furthermore, while all untreated cells were Ki67 positive (Fig. S5B), upon treatment the number of Ki67 positive cells were only slightly decreased for 4T1, MC38, CT26, PC9 and SKBr3 cells, while no HCT116 and only a few A549 cells were Ki67 positive (Fig. 4B,C).

In the three cell lines with the highest proliferation rate during MAPKi 4T1, MC38, and SKBr3, we observed that when MEKi and p38i were combined, a significant decrease in proliferation was observed when compared to MEKi alone demonstrating that the adaptive activation of p38 potentially leads to increased tumor cell growth during MEKi (Fig. 4D). Together, these data suggest that sustained activation of p38 leads to both decreased sensitivity toward MAPKi and increased expression of CD73, and thus, this pathway activation might drive a more aggressive and immunosuppressive phenotype.

### 3.6 Anti‐CD73 antibodies increase the antitumor effect of MEKi in a syngeneic mouse model

**Fig. 5:**
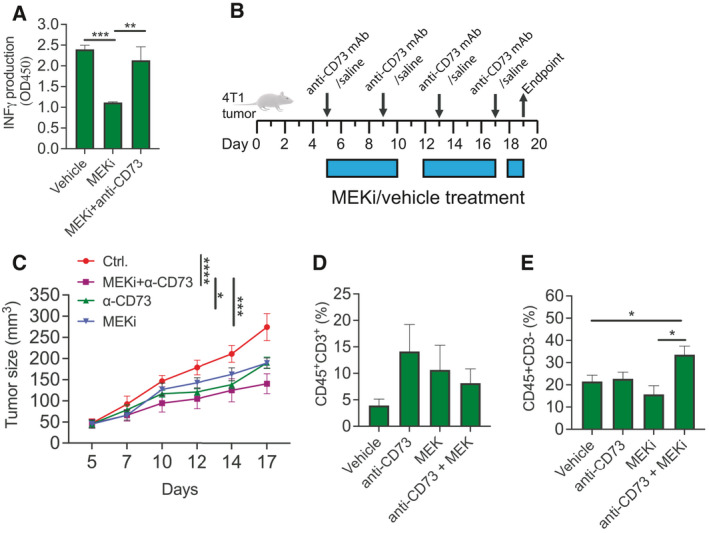
The functional impact of MEKi‐induced CD73 expression on T cells and tumor growth *in vivo* (A) IFNγ production by T cells exposed to CM from tumor cells treated with either vehicle, MEKi alone, or MEKi in combination anti‐CD73 antibodies for 5 days. *Asterisks* indicate significant differences using two‐tailed *t*‐test (**P < 0.01 and ***P < 0.001). (B) Scheme showing treatment schedule. (C) 4T1 cells were transplanted in immune competent mice. Starting on day 5 when tumors reach an average tumor volume of 50 mm^2^, mice were randomized to receive vehicle, daily MEKi (1 mg·kg^−1^), anti‐CD73 antibody (TY/23, days 5, 9, 13, and 17), or a combination of anti‐CD73 antibody and MEKi. *Asterisks* indicate significant differences in tumor size using mixed linear effect models for the drug combination‐treated mice compared to cells treated with either drug alone or control (*P < 0.05, ***P < 0.001, and ****P < 0.0001). (D–E) Tumors were harvested, digested to single‐cell suspensions, and analyzed by flow cytometry to determine the percentage of infiltrating immune cells. Asterisks indicate significant differences in one‐way ANOVA test with Bonferroni multiple comparison test between the different treatment groups. *P < 0.05.

Next, we investigated whether the increased CD73 expression following RAS‐MAPK pathway inhibition was sufficient to affect antitumor immunity. Using Interferon γ (IFNγ) secretion as a measure of T‐cell activation, we showed that T cells incubated in CM from 4T1 cells exposed to MEKi for 72h significantly decreased IFNγ production compared to T cells grown in CM from 4T1 cells not exposed to MEKi. The effect on T‐cell activation was rescued when CD73 was targeted using anti‐CD73 blocking antibodies (Fig. 5A). This suggests that increased CD73 expression and concomitant adenosine production by tumor cells have a potential biological impact on the TME. Next, we investigated a potential benefit of combining MEKi and targeted anti‐CD73 treatment in 4T1 tumor‐bearing mice (Fig. 5B). Using mixed linear effect models, we showed that tumor GR were significantly slower in mice treated with combined MEKi and anti‐CD73 antibody (GR = 7.91) than vehicle (GR = 18.36, *P* < 0.0001), anti‐CD73 antibody alone (GR = 10.97, *P* < 0.02), and MEKi alone (GR = 12.40, *P* < 0.001), respectively. This demonstrates an augmented effect of coblockade of CD73 and MEKi (Fig. 5C). We then analyzed the immune cell composition within the tumors, and while no difference in the numbers of tumor‐infiltrating lymphocytes (CD45+CD3+) was observed between the different treatment arms (Fig. 5D), there was a statistically significant enrichment of CD45^+^CD3^−^ cells in tumors treated with the MEKi/anti‐CD73 antibody combination compared to those treated with vehicle and MEKi alone (Fig. 5E). This suggests that combining MEKi and anti‐CD73 antibody exclusively attracts a population of CD45^+^CD3^−^ immune cells with antitumor effects. Collectively, our data demonstrate that MEKi potentially has a negative effect on the tumor immune microenvironment that could be prevented by cotreatment with anti‐CD73 antibodies.

## 4 Discussion

Changes in the TME, including the composition of immune cells and their activity, during the selective pressure of RAS‐MAPK‐targeted treatment play an important role in diminished treatment response and development of resistance in cancer patients. Intriguingly, our study demonstrates that RAS‐MAPK‐targeted treatment induces nongenomic adaptive bypass mechanisms that activate alternative kinase pathways leading to CD73 upregulation and thus promote immunosuppressive changes in the TME. In a recent study, it was shown that while CD73 was unchanged following EGFR‐TKI treatment, when all patients in the study were included in the analysis, increased CD73 expression was observed in the PD‐L1 high subgroup [32]. Together, this indicates that the link between RAS‐MAPK‐targeted treatment and CD73‐mediated alterations in the TME in a subset of MAPKi‐treated patients is a more general phenomenon across different cancer types. On the other hand, we also observed patients as well as cell lines that exhibited decreased CD73 expression following RAS‐MAPK‐targeted treatment, supporting recent studies showing that melanoma patients experienced decreased CD73 expression during BRAF treatment [18]. However, in that study, a subset of patients biopsied more than a week after BRAFi withdrawal also exhibited increased or steady CD73 expression, although the importance of this was not emphasized. Interestingly, we observed that the upregulation of CD73 following MAPKi varied both between different cell line models and patients. While further studies are needed to address this, it might be due to differences in expression/activation of upstream kinases (e.g., Mitogen‐activated protein kinase kinase (MKK)3,6) and/or inactivating phosphatases (e.g., dual‐specificity phosphatase (DUSP)s) and transcriptional regulators (e.g., transcription factors or epigenetic factors regulating chromatin structure). Collectively, this highlights the complexity of CD73 regulation during RAS‐MAPK‐targeted treatment and the importance of investigating the changes in expression in the individual patients. Furthermore, our data illustrate that in both the cell lines and the clinical paired before/during treatment samples, the expression of cyclin D1 (a classical RAS‐MAPK‐regulated transcriptional target [26]) was decreased following RAS‐MAPK inhibition, indicating that CD73 upregulation is compensatory in cancer cells during effective RAS‐MAPK kinase pathway inhibition.

Nongenomic adaptive bypass mechanisms that activate alternative pathways are likely to play an important role in escape from a particular targeted treatment. Adaptive crosstalk between the RAS‐MAPK and the p38 pathway has previously been suggested [33, 34, 35], and the MEK‐ERK1/2 pathway has been shown to be activated upon p38 inhibition, which promoted cancer cell survival. While four p38 isoforms exist, the demonstrated effect of p38α‐ and p38β‐specific inhibitors in our study suggests that p38α and p38β play a dominant role in the MAPKi‐induced CD73 upregulation. Both p38α and p38β can be activated by the same MAP2K isoform (MKK6) and have redundant functions and also activate similar downstream kinases [36]. In addition, an inhibitor targeting only p38α (ralimetinib) and knockdown of p38α (MAPK14) showed no or modest effects, suggesting that both p38α and p38β are involved in the upregulation of CD73, and when p38α is inhibited, p38β continues to drive the signaling pathway. We also evaluated previously identified regulators of CD73 including c‐JUN and CREB [17, 31], neither of which was shown to mediate CD73 upregulation. However, several other transcription factors have also been demonstrated to bind the CD73 promoter, including SP1, AP1, HIF1, and ATF‐1/2 [37, 38]. Further studies using ChIP‐PCR might identify binding of these factors to the CD73 promoter following MEKi‐induced p38 activation. Furthermore, we observed that sustained p38 activation mediated decreased MAPKi sensitivity and CD73 upregulation and thus might promote the development of more aggressive and immunosuppressive cancer cells. To this end, we extended an analysis of an existing dataset [22] and found that the median PFS was significantly shorter in cetuximab‐treated patients with high CD73 vs. low CD73 gene expression. In agreement with our findings, another study identified the CD73 gene, NT5E, among a set of 110 candidate genes for which higher expression was associated with decreased likelihood of disease control in metastatic colorectal cancer patients treated with cetuximab monotherapy [39]. In contrast, Cushman *et al*. identified CD73 gene expression as being beneficial to cetuximab treatment in combination with chemotherapy [40], but since CD73 expression has been shown to be upregulated following chemotherapy and also linked to chemotherapy resistance [31], this may complicate the evaluation of the effect of CD73 expression on cetuximab response. Recently, it was shown that infiltration of T cells, including CD8^+^ and effector memory CD4^+^, was significantly increased in tumors that responded to cetuximab, and this increase was independent of tumor mutation load or neoantigen burden [13], suggesting that T‐cell infiltration was not due to immunogenic cell death following cetuximab exposure. The immunosuppressive role of CD73, which increases the level of adenosine in tumors, is now well established [14, 15, 41, 42], and T‐cell infiltration may thus be decreased in the high CD73‐expressing tumors and so might contribute to the reduced response to cetuximab.

Finally, we evaluated the pathophysiological role of elevated levels of CD73 following MEKi treatment. The negative impact of increased CD73 expression following MEKi exposure on T‐cell activation exemplifies the potential clinical problem of MEKi‐induced modulation of the TME. Furthermore, our in vivo study provided rationale for targeting CD73 using antibodies in combination with MEKi to increase the antitumor effect of this treatment. Intriguingly, we observed a substantial increase of CD45^+^ CD3^−^ immune cells in tumors treated with the combination of MEKi and anti‐CD73 antibodies compared to those receiving either treatment alone, suggesting that the combination synergizes in the recruitment and potential activation of a CD45^+^ CD3^−^ immune cell population that facilitates antitumor activity. A different strategy to overcome MEKi resistance could potentially involve targeting p38 or another regulator in the activated resistance pathway. This might have a potentially broader effect that includes targeting the continued proliferation and increased adenosine production during MEKi treatment, as supported by our in vitro studies with combined MEKi and p38i. Interestingly, however, although the anti‐CD73 antibody TY/23 has been shown to inhibit primary tumor growth in a adenosine‐dependent manner [14], TY/23 compared to the small molecule inhibitor of CD73 (APCP) was superior in combination with the A2AR antagonist (A2ARi) with regard to metastatic control [16, 43]. This indicates that the activity of TY/23 is not solely dependent on targeting the enzymatic activity of CD73. To that end, a recent study has shown that the effect of TY/23 was dependent on a FcR‐positive CD11b^+^GR1^hi^ immune population in the pulmonary TME that were identified as neutrophils [16]. This population might correspond to the CD45^+^CD3^−^ population we identified in the combined MEKi and TY/23 treatment group. Taken together, this suggests that increased tumor CD73 following MEKi might increase FcR‐mediated recruitment of CD45^+^CD3^−^ cells, leading to the observed augmented effect of anti‐CD73 antibody combined with MEKi, while the effect of limiting adenosine might only partly contribute to the effect of TY/23 in our model.

## 5 Conclusions

**Fig. 6:**
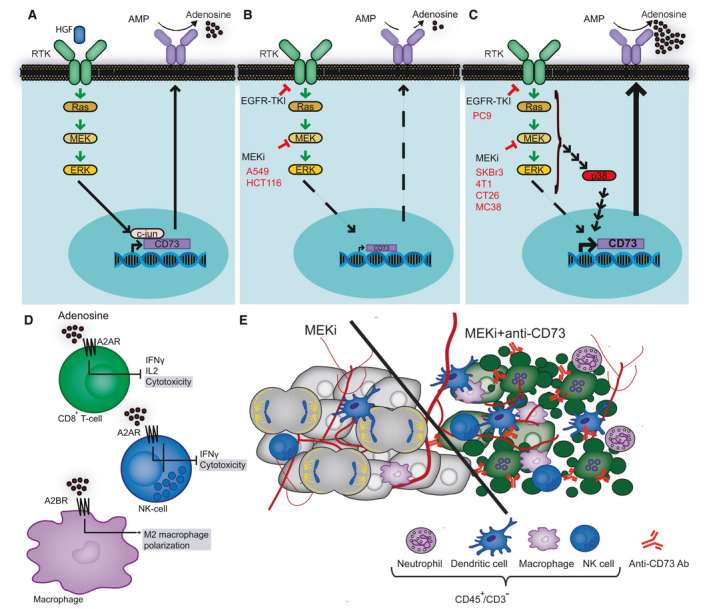
Schematic presentation of the mechanism underlying RAS‐MAPK inhibition‐induced upregulation of CD73 expression in a subset of cancer cells lines. (A) CD73 expression is stimulated by strong activation of the RAS‐MAPK pathway through HGF activation of the RTK. (B) Exposure to MEKi in HCT116 and A549 cells decreases the expression of CD73. (C) Exposure to EGFR‐TKI in PC9 and MEKi in SKBr3, 4T1, CT26, and MC38 cells activates p38, which subsequently induces CD73 expression. (D) CD73 produced adenosine suppresses the cytotoxicity of both CD8^+^ and NK cells and induces polarization of M2 macrophages. (E) Co‐administration of anti‐CD73 antibodies and MEKis might accelerate the recruitment of innate immune cells to control tumor growth. RKT, receptor tyrosine kinase and HGF, hepatocyte growth factor.

In conclusion, this study provides evidence of a rapid adaptive bypass mechanism that upon MAPK pathway blockade can shift the transcriptional control, leading to the unexpected increase of CD73 expression (Fig. 6A–C). This upregulation of CD73 supports an immune suppressive TME and potentially facilitates development of resistance toward MAPKi‐targeted treatment (Fig. 6D). Importantly, however, anti‐CD73 antibodies in combination with MEKis might accelerate the recruitment of a CD45^+^ CD3^−^ immune cell population to control tumor growth (Fig. 6E). Further studies are needed to address the precise composition and function of this immune cell population that may include cell types affected by adenosine (Fig. 6D) or expressing Fc receptors such as macrophages, NK cells, dendritic cells, and neutrophils (Fig. 6E).

## Conflict of interest

The authors declare no conflict of interest.

## Author contributions

MGT and HJD conceptualized the study, wrote the original draft, and acquired the funding. MGT and OLG performed the methodology. MGT, OLG, HV, MFG, and HJD involved in the investigation. HJD provided the resources and supervised the study. All authors wrote, reviewed, and edited the manuscript.

### Peer Review

The peer review history for this article is available at https://publons.com/publon/10.1002/1878‐0261.13046.

## Supporting information


**Fig. S1.** The distribution of expression values for the selected clinical samples.
**Fig. S2.** CD73 baseline expression and expression changes following exposure to various small molecule inhibitors.
**Fig. S3.** Densitometry quantification of p‐p38.
**Fig. S4.** Combination of RAS‐MAPK inhibitors and p38 inhibition.
**Fig. S5.** Proliferation assays of RAS‐MAPK inhibitor and control treated tumor cells.
**Table S1.** Univariate analysis of KRAS mutation status and CD73 gene expression in CRC patients.

## Data Availability

The data that support the findings of this study are openly available in [Gene Expression Omnibus] at [https://www.ncbi.nlm.nih.gov/geo/query/acc.cgi?acc=GSE114082], reference number [GSE114082]; [Gene Expression Omnibus] at [https://www.ncbi.nlm.nih.gov/geo/query/acc.cgi], reference number [GSE99898]; [Gene Expression Omnibus] at [https://www.ncbi.nlm.nih.gov/geo/query/acc.cgi], reference number [GSE98399]; and [Gene Expression Omnibus] at [https://www.ncbi.nlm.nih.gov/geo/query/acc.cgi], reference number [GSE5851].
