# Supplementary material for: Sustained compensatory p38 MAPK signaling following treatment with MAPK inhibitors induces the immunosuppressive protein CD73 in cancer: combined targeting could improve outcomes
Source: Mol Oncol. 2021 Jul 16;15(12):3299–316. doi: 10.1002/1878-0261.13046 (PMC8637576; doi:10.1002/1878-0261.13046)
Supplement: Supplementary file 1 — Fig. S1. The distribution of expression values for the selected clinical samples. Fig. S2. CD73 baseline expression and expression changes following exposure to various small molecule inhibitors. Fig. S3. Densitometry quantification of p‐p38. Fig. S4. Combination of RAS‐MAPK inhibitors and p38 inhibition. Fig. S5. Proliferation assays of RAS‐MAPK inhibitor and control treated tumor cells. Table S1. Univariate analysis of KRAS mutation status and CD73 gene expression in CRC patients. [file MOL2-15-3299-s001.pdf]

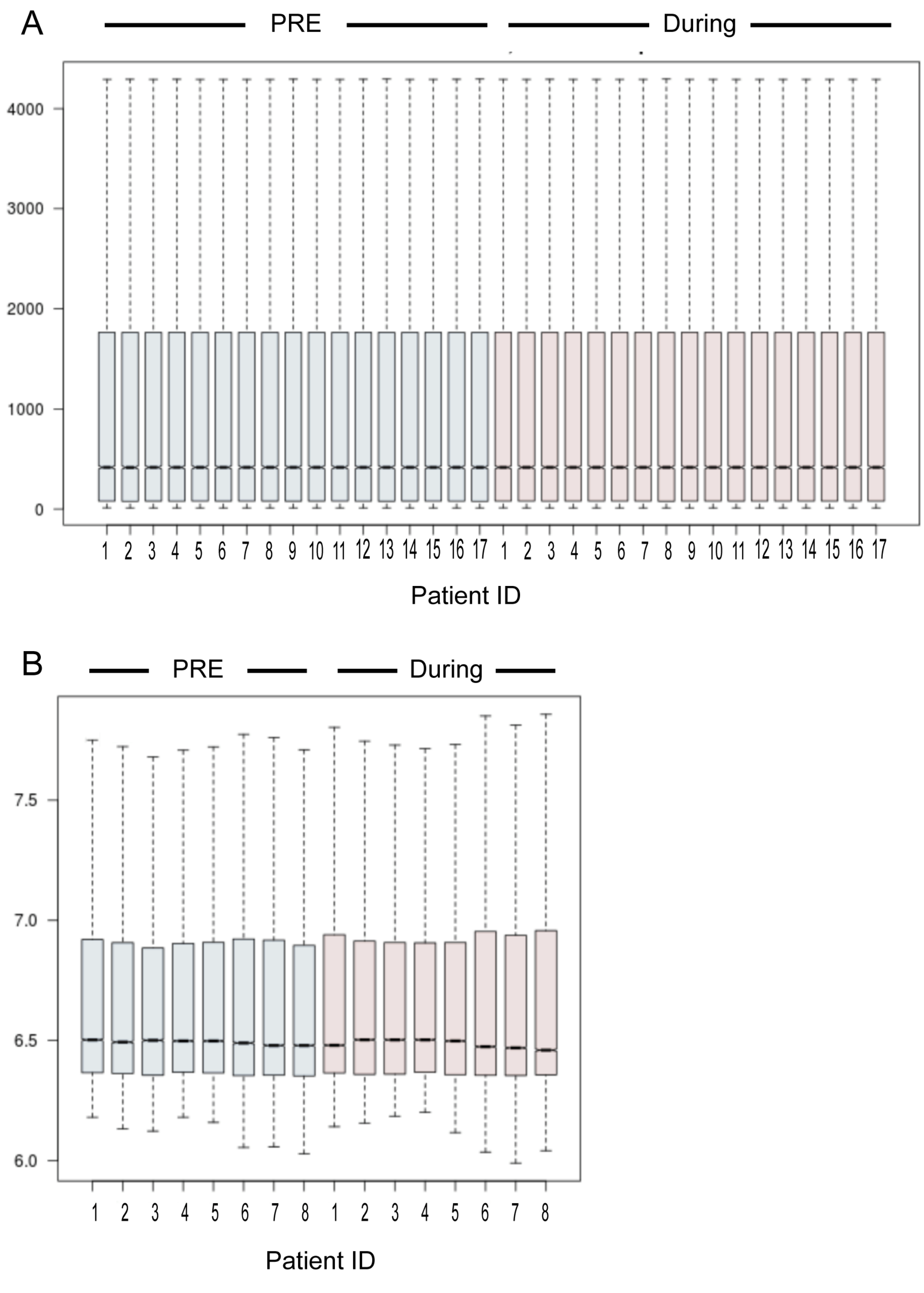

Supplementary figure 1. The distribution of expression values for the selected clinical samples. The median-centered values indicate the data are normalized and cross-comparable. **(A)** breast cancer patients (GSE114082) and **(B)** melanoma patients (GSE99898).

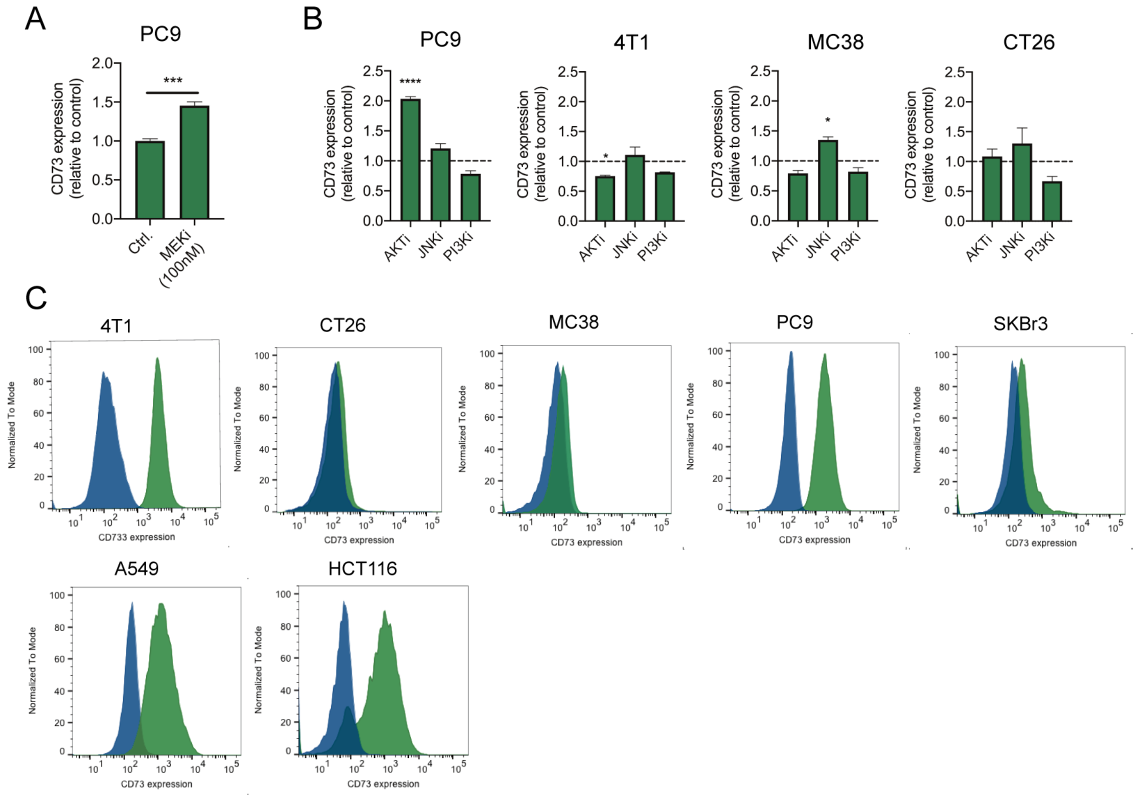

Supplementary figure 2. CD73 baseline expression and expression changes following exposure to various small molecule inhibitors. (A) PC9 cells were exposed to MEKi for 72h and CD73 protein expression was analyzed by flow cytometry. **(B)** PC9, 4T1, CT26 and MC38 cells were exposed to AKT inhibitor (2.5 µM), JNK inhibitor (1 µM) or PI3K inhibitor (1 nM) for 72h and CD73 protein expression was analyzed by flow cytometry. **(C)** CD73 expression (Green) in PC9, SKBr3, A549, HCT116, 4T1, CT26 and MC38. The corresponding cells stained with only secondary Alexaflour488 anti-IgG is shown in blue. The shift in staining intensity corresponds to level of CD73 expression. Results are shown as mean ± SD and represents three replicates. *Asterisks* indicate significant differences in a one-way ANOVA test with Bonferroni multiple comparison test at the same time point. (*$p < 0.05$; **$p < 0.01$, ***p<0.001, ****p<0.0001)

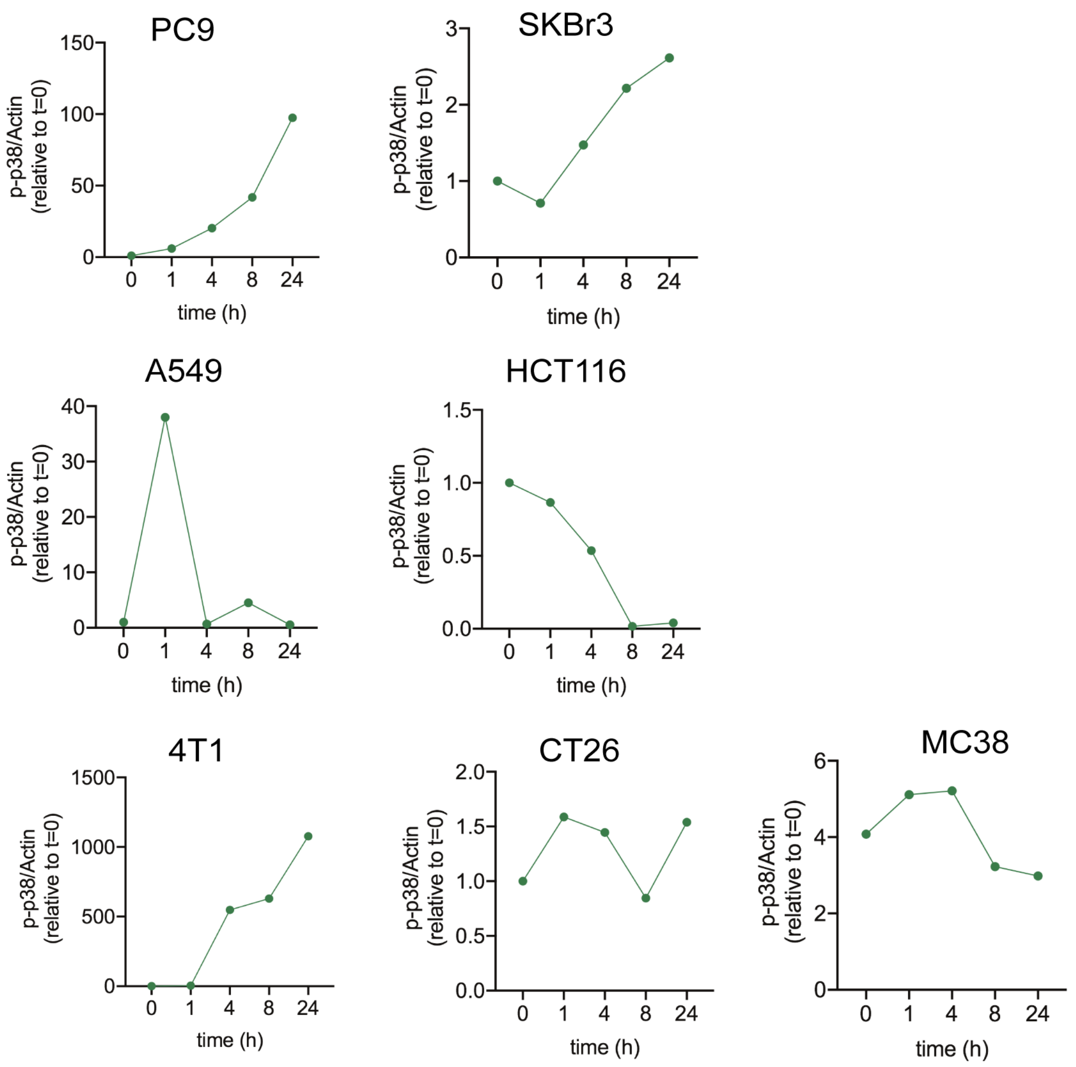

Supplementary figure 3. Densitometry quantification of p-p38 Densitometry quantification of p-p38. Band intensity values from the Western Blots in Figure 3 of the target proteins were normalized to the band intensity of the corresponding actin band.

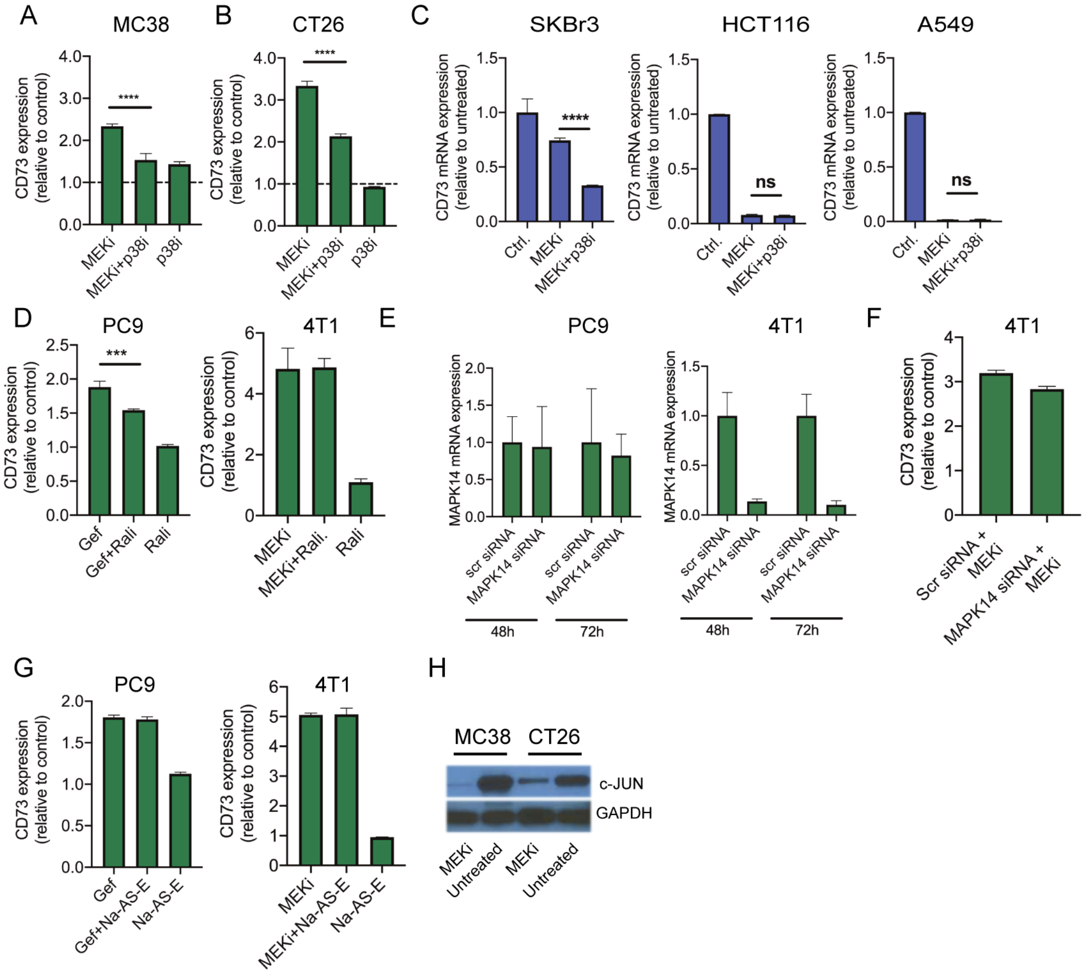

Supplementary figure 4. Combination of RAS-MAPK inhibitors and p38 inhibition. (A) MC38 and **(B)** CT26 cells were exposed to MEKi (100 nM) and p38i (10 μM), alone or in combination for 72h, at which CD73 expression was analyzed by FACS **(C)** SKBr3, HCT116 and A549 cells were exposed to MEKi (100 nM) alone or in combination with p38i (10 μM), for 72h, at which time point CD73 expression was analyzed by RT-PCR. **(D)** PC9 and 4T1 cells were exposed to Gefitinib (Gef, 100 nM) and MEKi, respectively alone or in combination with the p38α specific inhibitor Ralimetinib (Rali, 1μM) for 72h, at which time point CD73 expression was analyzed by flowcytometry. **(E)** PC9 and 4T1 cells were transfected with MAPK14 siRNA and at the indicated times the expression of MAPK14 was evaluate by RT-PCR. **(F)** 4T1 cells were transfected with MAPK14 siRNA. Following 48h, cells were treated with MEKI (100 nM) for 72h at which time point CD73 expression was analyzed by flowcytometry. **(G)** PC9 and 4T1 cells were exposed to Gefitinib (Gef, 100 nM) and MEKi, respectively, alone or in combination with Napthol-AS-E (100 nM) for 72h, at which time point CD73 expression was analyzed by flowcytometry**. (G)** Western blot analysis of c-jun expression in MC38 and CT26 cells exposed to MEKi for 72h. Results are shown as mean ± SD and represents three replicates. *Asterisks* indicate significant differences in one-way ANOVA test with Bonferroni multiple comparison test for the drug combination-treated cells compared to cells treated with either drug alone. (*p < 0.05; **p < 0.01, ***p<0.001, ****p<0.0001)

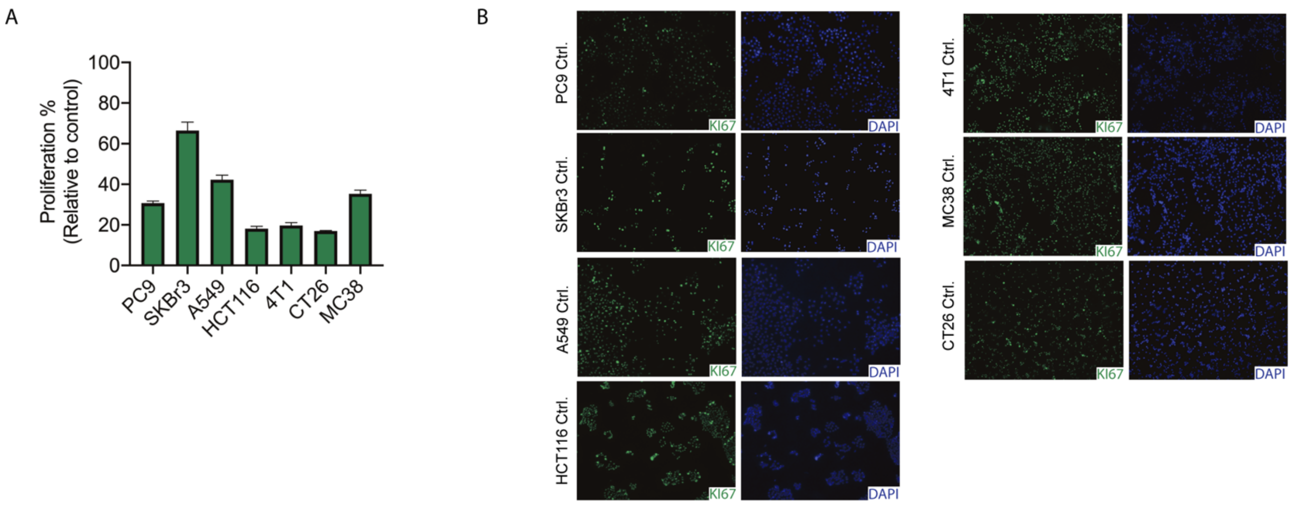

Supplementary figure 5. Proliferation assays of RAS-MAPK inhibitor and control treated tumor cells. **(A)** Cells were seeded at low density. PC9 was exposed to EGFR-TKI (100 nM) and SKBr3, HCT116, A549, 4T1, MC38, CT26 were exposed to MEKi (100nM). Tumor cell growth was determined at the indicated times using crystal violet staining and shown relative to untreated control. Results are shown as mean ± SD and represents three replicates. **(B)** Cells were seeded at low density and grown without any treatment. After 72h cells were stained for ki67 expression and counterstained with DAPI.

Univariate analysis			
	Hazard Ratio	95% CI	P
CD73 expression	2.02	1.23-3.30	0.005
KRAS mutation status (mutated vs. WT)	0.68	0.41-1.14	0.154

Supplementary Table 1. Univariate analysis of KRAS mutation status and CD73 gene expression in CRC patients. Association with PFS of metastatic colorectal cancer patients treated with cetuximab monotherapy.
